# New Antioxidant Drugs for Neonatal Brain Injury

**DOI:** 10.1155/2015/108251

**Published:** 2015-01-05

**Authors:** Maria Luisa Tataranno, Serafina Perrone, Mariangela Longini, Giuseppe Buonocore

**Affiliations:** Department of Molecular and Developmental Medicine, University of Siena, 53100 Siena, Italy

## Abstract

The brain injury concept covers a lot of heterogeneity in terms of aetiology involving multiple factors, genetic, hemodynamic, metabolic, nutritional, endocrinological, toxic, and infectious mechanisms, acting in antenatal or postnatal period. Increased vulnerability of the immature brain to oxidative stress is documented because of the limited capacity of antioxidant enzymes and the high free radicals (FRs) generation in rapidly growing tissue. FRs impair transmembrane enzyme Na^+^/K^+^-ATPase activity resulting in persistent membrane depolarization and excessive release of FR and excitatory aminoacid glutamate. Besides being neurotoxic, glutamate is also toxic to oligodendroglia, via FR effects. Neuronal cells die of oxidative stress. Excess of free iron and deficient iron/binding metabolising capacity are additional features favouring oxidative stress in newborn. Each step in the oxidative injury cascade has become a potential target for neuroprotective intervention. The administration of antioxidants for suspected or proven brain injury is still not accepted for clinical use due to uncertain beneficial effects when treatments are started after resuscitation of an asphyxiated newborn. The challenge for the future is the early identification of high-risk babies to target a safe and not toxic antioxidant therapy in combination with standard therapies to prevent brain injury and long-term neurodevelopmental impairment.

## 1. Introduction

The most common causes of neonatal brain injury in developing countries are extreme prematurity, neonatal stroke, and hypoxic-ischemic encephalopathy (HIE) [1]. Extreme prematurity leads to an increased risk of death or long-term neurodevelopmental impairment including cerebral palsy (CP) [2–4], but it also contributes to half of pediatric costs for health care. The second and third trimesters represent the most important period for brain development, with a rapid increase in size, shape, and complexity [5]. Hypoxia-ischemia, inflammation, and oxidative stress can lead to an interruption of normal brain development especially during this delicate period resulting in structural, biochemical, and cell-specific injury [6]. The preoligodendrocytes, which emerge and mature between 24 and 32 weeks of development, are particularly susceptible to injury such as intracranial hemorrhage, periventricular leukomalacia, and other inflammatory conditions, and this damage can result in white matter injury [7, 8]. Oxidative stress (OS) plays a fundamental role in early injury, along with other mechanisms such as excitotoxicity, to the neonatal brain. OS in vivo is a degenerative process due to overproduction of free radicals (FRs) (reactive oxygen species and reactive nitrogen species) and propagation of their reactions. These FRs include superoxide anion (O_2_^−^), hydroxyl radical (OH), singlet oxygen (^1^O_2_), and hydrogen peroxide (H_2_O_2_). FRs cause damage to lipids, protein, and DNA, initiating a cascade that results in cell death [9]. OS exists and tissue damage is possible when there are low levels of antioxidants or increased FR activity [10]. Brain cells death at any age is primarily due to hypoxia and energy depletion, followed by reperfusion and FR overproduction. Excitotoxicity and nitric oxide (NO) production are responsible of secondary energy failure and delayed death. All these deleterious biological events trigger the inflammatory response with cytokine production which plays a major role in cell damage and loss. Local microglia are activated, producing proinflammatory cytokines such as tumor necrosis factor- (TNF-) alpha, interleukin- (IL-) 1b, and IL-6, as well as glutamate, FR, and NO, and are the main immunocompetent cells in the immature brain [11].

Newborns and particularly preterm infants are at high risk for OS and damage due to their organs' structural and functional immaturity with the lack of antioxidant enzyme production, the overloading of aerobic metabolism with rapidly growing energy demand, and the presence of conditions leading to increased free iron levels with excessive FR production.

Neonatal plasma has profoundly disturbed antioxidant profiles with low levels of gluthatione peroxidase activity, superoxide dismutase, *β*-carotene, riboflavin, *α*-proteinase, vitamin E, selenium, copper, zinc, ceruloplasmin, transferrin, and other antioxidant plasma factors [12].

In the developing fetus, hypoxia results in increased anaerobic metabolism, leading to a rapid rise in levels of lactic acid and reduced forms of electron transport chain components in mitochondria, with subsequent FR overproduction [13]. Other mechanisms contributing to reactive oxygen FR formation and membrane lipid peroxidation include phagocyte activation, metabolism of arachidonic acid through the cyclooxygenase and lipoxygenase pathways, reactions catalysed by increased free intracellular Fe^++^, and increased xanthine oxidase activity as a result of increased degradation of ATP [14]. Excess of free iron and deficient iron/binding metabolising capacity are additional features favouring OS in the premature infants [15]. The properties and complex role of FR in the development of diseases make antioxidant therapy very difficult to be realised particularly because a critical aspect of protection is the prevention of the hypoxic-ischemic insult which often happens already in utero; thus prevention of intrauterine asphyxia is of great relevance.

Insights into the biochemical and cellular mechanisms of cellular injury with perinatal hypoxic-ischemic-reperfusion insults provide a rational basis for formulation of interventions to interrupt those mechanisms and thereby prevent or ameliorate the injury.

**Figure 1:**
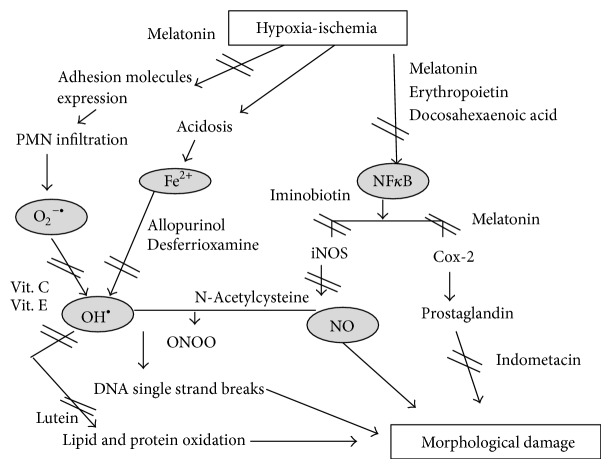
Oxidative stress pathways and the possible antioxidant drugs targets.

Antioxidant substances may act at different phases of the injury, decompartmentalizing metal complexes, limiting FR production, modifying antiradical defenses, enhancing intracellular or extracellular antioxidant levels, and incorporating lipophilic antioxidants into membranes or scavenging superoxide [16]. Some drugs may inhibit phagocyte activation or xanthine oxidase and arachidonic acid metabolism or directly scavenge FR or repair FR-induced membrane injury, like calcium antagonists and beta blockers. Elimination of transition metals and especially nonprotein bound iron (NPBI) is crucial for interrupting the formation of FR [17] Figure 1.

Although many antioxidant agents have been shown to be protective in animal models of hypoxia-ischemia, only few substances have been used in pilot studies for newborns. This paper highlights some of the important future therapeutics for neuroprotection and describes which pharmacological interventions can be considered to reduce brain injury in the neonatal high risk population.

## 2. Vitamins C and E

Vitamins C and E are essential nutrients and are considered the most important antioxidants obtained through the diet. The antioxidant actions of vitamin E (*α*-tocopherol and *β*-tocopherol) are due to its ability to be incorporated into biological membranes to stabilize and protect against lipid peroxidation [18], while the antioxidant properties of vitamin C (ascorbic acid) arise from the ability to act as electron donor, thereby preventing other agents from becoming oxidized and quenching overproduction of FR. Studies regarding the protective benefits of vitamins C and E in the perinatal period are limited. Some in vitro evidences suggest that, in adult and fetal rat brain cultures, vitamin E is able to decrease lipid peroxidation and to increase survival and neuritic extension of neurons [19, 20]. In vivo, prophylactic administration of vitamin E before hypoxia-ischemia is able to decrease the incidence of IVH [21]. Protective effects on retinopathy of prematurity (ROP) have also been reported with a reduction of ROP III+ from 5.3 to 2.8% [22]. In a mouse model of Down syndrome, *α*-tocopherol suppresses lipid peroxidation in the hippocampus and ameliorates behavioral and cognitive impairments [23]. *α*-tocopherol has also been shown to have anti-inflammatory properties. Administration of *α*-tocopherol, particularly in large doses, has been shown to decrease the release of proinflammatory cytokines from cell lines exposed to lipopolysaccharide [24].

Ascorbate deficiency in the postnatal mouse brain (in the presence of normal GSH levels) leads to diminished motor functions, yet an exaggerated response to a dopaminergic agonist [25]. Ascorbate antioxidant effects are enhanced in conjunction with vitamin E. When vitamin E is oxidized, it forms *α*-tocopherol radical which is harmful, but vitamin C is able to mediate the return of *α*-tocopherol radical to *α*-tocopherol, thus regenerating *α*-tocopherol concentrations in plasma [26]. In support of these findings, a study of transient intrauterine ischemia in pregnant rats showed that either vitamin E or vitamin C treatment alone, started before the ischemic insult, was able to decrease oxidative mitochondrial impairment in the fetal brain, but the improvement was greater when vitamins were administered together [27].

On a cautionary note vitamin C has both prooxidant or antioxidant effects in vitro; it is able to inhibit protein synthesis and induce late neuronal death [28–30]. Similarly, vitamin E may induce apoptosis in absence of OS [31], potentially limiting its protective effects only to situations when OS is established. It is hypothesized that protecting the fetus through the pretreatment of the mother could in itself be beneficial and without any additional risk burden on either the mother or her baby, although the possibility of toxicity of these agents in absence of OS was also postulated.

## 3. Inhibitors of NOS

Nitric oxide (NO) is a free radical that is formed in high concentrations during and after hypoxia-ischemia. Three enzymes especially upon reperfusion and reoxygenation after perinatal HI can catalyze the formation of NO: neuronal NOS (nNOS), inducible NOS (iNOS), and the endothelial NOS (eNOS) [32] that are all expressed in the brain in neurons, astrocytes, and endothelial cells and can be induced in microglia. eNOS is thought to have a neuroprotective function* via *enhancing perfusion of the brain if necessary [33, 34].

NO can react with superoxide to form peroxynitrite, which can cause nitration of proteins, predominantly on tyrosine residues contributing to further damage to brain tissue [35]. Selective inhibition of nNOS and iNOS with the nNOS inhibitor, 7-nitroindazole, and the iNOS inhibitor aminoguanidine have been proved to be promising as neuroprotectants in neonatal rats [36–38].

The pharmacologic inhibition of nNOS, or its genetic deletion, confers neuroprotection in animal models of transient cerebral ischemia [38, 39]. Iminobiotin inhibits both the neuronal and inducible isoforms of nitric oxide synthase. Otherwise, in vivo, it provides neuroprotection probably hindering apoptotic pathways. Nijboer et al. demonstrated that treatment with 2-iminobiotin provided gender specific long- and short-term neuroprotection in female newborn rats with hypoxia-ischemia via inhibition of the cytochrome c-caspase 3 neuronal death pathway [40]. However, only female and not male animals were protected against post-HI reperfusion damage to the brain [41, 42]. Moreover, the existing evidence suggests that the in vivo neuroprotective effect of 2-iminobiotin was not dependent on nNOS/iNOS inhibition [41, 43]. The exact mechanism of action of 2-IB remains to be determined, but it is clear that in females neuroprotection is associated with reduced activation of the apoptotic pathways.

## 4. Allopurinol

Allopurinol and its metabolite oxypurinol are inhibitors of xanthine oxidase, the enzyme involved in superoxide production especially during reperfusion damage. Allopurinol has also additional effects, directly scavenging the toxic hydroxyl free radical and mainly chelating the nonbound protein iron (NBPI), particularly at high doses [44]. Allopurinol is converted into oxypurinol, which crosses the blood brain barrier more easily. Palmer et al. were the first to recognize the neuroprotective properties of allopurinol in a neonatal rat model of HI brain injury [45]. The data of this study were promising, although another study was less positive on the neuroprotective potential of allopurinol [46]. In asphyxiated infants who received allopurinol, NO serum level decreased significantly from 24 hours to 72–96 hours after birth [47]. The treated newborns had also better neurodevelopmental outcomes. In other studies in humans allopurinol was not seen to improve short-term or long-term outcomes after birth asphyxia [48]. In a pilot study, Torrance et al. administrated to 53 pregnant women in labor (54 fetuses with a gestational age >36 weeks and signs of fetal hypoxia) 500 mg of allopurinol or placebo intravenously. They found a reducing effect on biomarkers of neuronal damage and NPBI [49]. It is possible that allopurinol has no positive effect when administered late and at low doses [50]. It was hypotesized that the drug needed to be administrated before the start of reperfusion injury, so trials are now underway to evaluate efficacy when given to mothers who have fetuses suspected of intrauterine hypoxia [8]. In a randomized, double-blind, placebo-controlled multicenter study that is now in progress, intravenous allopurinol is being given antenatally with the primary outcome being serum brain damage markers (S100b) and oxidative stress markers (isoprostanes and so forth) in umbilical cord blood, while secondary outcome measures are neonatal mortality, serious composite neonatal morbidity, and long-term neurologic outcome [51]. There is now a randomized, placebo-controlled, double-blinded parallel group comparison study of hypothermia and allopurinol ongoing (the European ALBINO Trial). Allopurinol is being given twice: 30 minutes after birth and then 12 hours later, in addition to hypothermia in moderate to severe HIE. Outcomes will be assessed at 2 years of life. Kaandorp and colleagues found no differences in long-term outcome between the allopurinol-treated infants with moderate to severe birth asphyxia and controls. However, subgroup analysis of the moderately asphyxiated group showed significantly less severe adverse outcome in the allopurinol-treated infants compared with controls (25% versus 65%; RR 0.40, 95% CI 0.17 to 0.94), suggesting a protective effect of neonatal allopurinol treatment in moderately asphyxiated infants [52].

## 5. Erythropoietin

Erythropoietin (Epo) and its receptor (EpoR) are expressed in several types of cells including astrocytes and microglial cells of the developing central nervous system and are required for normal brain development [53]. The acute exposure to hypoxia upregulates the expression of EpoR on oligodendrocytes and neurons, without a similar increase in Epo expression [54]. The presence of unbound EpoR drives cells of neuronal and oligodendrocyte lineage to apoptosis, whereas ligand-bound EpoR activates survival signaling pathways [55]. The signalling cascade activated by Epo is able to block early mechanisms of brain injury by its antiinflammatory, antiexcitotoxic, antioxidant, and antiapoptotic effects on neurons and oligodendrocytes [56–58]. Erythropoietin (Epo) is directly involved in the prevention of OS with generation of antioxidant enzymes, inhibition of nitric oxide production, and decrease of lipid peroxidation [59]. Moreover, Epo can decrease the production of proinflammatory cytokines and of the associated apoptotic injury, as it happens in adult stroke and neonatal models of hypoxia-ischemia treated with rEPO [57, 60]. Higher circulating Epo concentrations might produce more benefits than lower concentrations [61]. In addition the beneficial effects are dose dependent, with multiple doses being more effective than single doses [62] even when initiated 48 to 72 hours after injury reducing neuronal loss and learning impairment [63, 64]. Epo is now under investigation for both term and preterm brain injuries, being effective in reducing brain injury in both groups and it is approved by the US Food and Drug Administration with a robust safety profile in neonates.

The specific effects of Epo in preoligodendrocytes may be most relevant to the white matter injury that characterizes preterm brain injury [8]. Treatment approaches should be differentiated in case of proven brain injury (HIE of term infants and IVH in preterm infants) and preventative strategies in preterm infants. In the first case (brain injury) a shorter duration of high-dose Epo is most appropriate, whereas a more prolonged treatment strategy that continues during the period of oligodendrocyte maturation is most likely to succeed for preventative purposes. Furthermore, this more prolonged treatment also decreases the availability and potential toxicity of free iron, caused by the erythropoietic effects of Epo, by increasing iron utilization [8].

In addition, the impossibility of Epo to cross the placenta makes the prenatal treatment unavailable even if some new studies suggest that a dose of 500 mg intravenous allopurinol rapidly crosses the placenta and provides target concentrations in 95% of the fetuses at the moment of delivery, which makes it potentially useful as a neuroprotective agent in perinatology [65, 66].

Doses of 1000 to 5000 U/kg were effective for neuroprotection in animal models of neonatal brain injury, improving both short-term and long-term structure and function [67]. Phase I/II trials on human newborns [68–70] suggested 1000 U/kg/dose as the optimal dose. Phases II and III studies are now ongoing in both preterm and HIE term infants. Epo is also being studied in combination with hypothermia for the treatment of HIE in term infants. Adjunctive use of Epo combined with hypothermia in nonhuman primates subjected to umbilical cord occlusion was showed to improve motor and cognitive responses, cerebellar growth, and diffusion tensor imaging measures, producing death/disability relative and absolute risk reduction [71, 72]. Conversely, in a rat model of hypoxia-ischemia, no significant benefit or, at least, only a borderline additive neuroprotective effect of EPO combined with hypothermia was observed [73, 74].

In various experimental models, Epo demonstrates a neuroprotective effect particularly after neuronal damage related to ischemia-reperfusion events. Early treatment after HI with a high dose of Epo (5000 U/kg) reduces tissue loss, preserving brain volume [62], and enhances neurogenesis, probably through a shift from astrocytic to neuronal cell fate [75].

A therapeutic strategy with lower multiple Epo doses, such as 1000 U/kg, did not result in significant neuroprotection from early neuronal damage, even when combined with deferoxamine, an iron chelator which has been shown to decrease OS [76].

A randomized prospective study reported that repeated, low doses (300 or 500 U/kg every day) of Epo were safe and resulted in improved neurological outcome for patients with moderate neonatal HIE at 18 months of age [77]. Recently, in an analysis of secondary outcomes of a randomized clinical trial of preterm infants, high-dose erythropoietin treatment (3000 IU/kg) within 42 hours after birth was associated with a reduced risk of brain injury on MRI [72].

A high survival rate with no or minor cerebral sequelae was observed in adult patients treated with hypothermia and early high doses of Epo therapy (40 000 IU) for the first 48 h, soon after resuscitation from cardiac arrest, in a small pilot study [78]. Safety concerns appeared from some adult Epo trials reporting adverse effects related to vascular thrombosis, intracerebral hemorrhage, and brain edema [79].

## 6. Albumin and Docosahexaenoic Acid (DHA)

Albumin is the main protein of plasma, representing about 60% of all plasma proteins. Marzocchi et al. demonstrated albumin carbonylation in newborns with higher NBPI levels and poor neurodevelopmental outcome [80]. Since NBPI may produce hydroxyl radicals through the Fenton reaction, the major target of OS induced by NBPI is its carrier, albumin. As albumin is a major extracellular antioxidant, its susceptibility to oxidation can be expected to decrease plasma antioxidant defenses and increase the likelihood of tissue damage due to OS in the newborn. Nitrated albumin was found significantly increased in patients who developed moderate or severe encephalopathy compared to those who had a normal neurological evolution or developed mild encephalopathy [81]. There is evidence that albumin significantly enhances neurological function and may decrease brain edema and infarction if administered 4 hours after ischemia occurrence in adult rats [82]. In clinical trials it was observed that administration of albumin may cause side effects on lungs [83]. To reduce this side effect, albumin in low doses may be administered in association with docosanoids. Docosanoids are derivates of docosahexaenoic acid (DHA), which is a major product of the oxidative lipid degradation of the membrane after cerebral ischemia; its bioproducts can inhibit the infiltration of leukocytes and reduce expression of NF-*κ*B. DHA treatment before the insult confers neuroprotection in a rat model of cerebral hypoxia-ischemia [84]. DHA improves function without affecting brain volume loss in a rat model of hypoxia-ischemia and inflammation induced perinatal brain injury [85].

## 7. Deferoxamine

Deferoxamine is a chelating agent and its target is the formation of hydroxyl radicals from free iron during reperfusion. Deferoxamine can cross the blood brain barrier and chelate NBPI, thus reducing the severity of brain injury and improving cerebral metabolism in animal models of hypoxia-ischemia [86]. Deferoxamine treatment in lambs limited the hypoxia-ischemia induced NBPI increase in plasma and in cortical tissue [87]. Negative effects on hemodynamics when administrated at high doses in preterm baboons have been observed [88]. Several experimental studies have been performed in newborn animals of various species including pigs and rats with positive results [89, 90]. Up to now, however, free ion chelators have never been tested in newborn babies to treat reperfusion/reoxygenation injury after perinatal HI.

## 8. Prostaglandins Inhibitors

The inhibition of prostaglandin production was hypothesized to be another important target to fight post-HI brain damage in the newborn. Indomethacin is a cyclooxygenase inhibitor and has been shown to reduce neonatal brain damage after perinatal HI in experimental studies [91]. Indomethacin is used in preterm babies to reduce or prevent the occurrence of periventricular/intraventricular hemorrhages [92, 93] and can reduce white matter injury in preterm infants [91, 94, 95]. It has not been experimented yet in the term infant to reduce reperfusion/reoxygenation injury of the brain after perinatal HI.

## 9. Magnesium Sulphate

Magnesium is essential for key cellular processes, like glycolysis, oxidative phosphorylation, proteins synthesis, and DNA and RNA aggregation [96]. Moreover magnesium can influence mechanisms implicated in cell death due to the production of proinflammatory cytokines during the inflammatory response and, through the noncompetitive voltage dependent inhibition of the NMDA-type glutamate receptor, it can reduce calcium entry in the cells leading to the prevention of the excitotoxic calcium induced injury [97]. Recent evidences demonstrate that fetal exposure to magnesium sulfate in women at risk for preterm delivery significantly reduces the risk of cerebral palsy without increasing the risk of death [98–100]. In adult rats with cerebral ischemia, magnesium sulfate was demonstrated to be more effective, increasing neuronal survival rate, than either treatment used alone [92]. Most of the positive evidence with magnesium sulfate is for the preterm population [93]; preclinical and clinical evidence for magnesium sulfate in term hypoxia-ischemia are less good than other approaches [101–103]; and term infants may have significant risk of hypotension at high doses [104]. Magnesium has other important side effects: it can provoke haemodynamic instability, bradycardia, and delayed intraventricular conduction, including complete atrioventricular block [105]. It appears that magnesium sulfate is ineffective in delaying birth or preventing preterm birth when used as a tocolytic [106]. Furthermore, high cumulative doses of magnesium sulfate may be associated with increased infant mortality [107]. The evidence to date confirms the efficacy of magnesium sulfate therapy for women with eclampsia and preeclampsia and for reduction of cerebral palsy in the setting of threatened preterm delivery. However, magnesium sulfate should not be used as a tocolytic for preterm labor. A recent review from Galinsky and colleagues suggested the inconsistent histopathological impact of treatment both before and after the hypoxic-ischemic insult, likely related to the lack of temperature control during and after HI, along with variability in the dose, timing of treatment, and survival time after the insult [108]. These findings strongly suggest that clinical trials of MgSO_4_ for encephalopathy at term would be premature, despite the very promising results from studies of global or focal ischemia in adult rodents. These observations suggest a need for further testing in animal models of HIE before magnesium should be considered for trials in humans as a potential adjunct therapy with hypothermia.

## 10. N-Acetylcysteine

N-Acetylcysteine (NAC) is a scavenger of oxygen radicals and restores intracellular glutathione levels, attenuating reperfusion injury and decreasing inflammation and nitric oxide (NO) production in adult models of stroke [109]. It has low toxicity and it is able to cross the placenta and the blood-brain barrier. In most experiments to evaluate neuroprotective effects of NAC, repeated administration has been used. In an animal model of hypoxia-ischemia, NAC treatment combined with systemic hypothermia prevented brain tissue loss, with increased myelin expression and improved the short-term functional outcomes of labyrinthine and cerebellar integration [110]. Consistently with this, Cakir et al. reported that after spinal cord ischemia, NAC and hypothermia alone were associated with limited protective effects, whereas the combination of NAC and hypothermia resulted in highly significant recovery of spinal cord function [111]. NAC may also have anti-inflammatory effects, by reducing intracerebral levels of tumor necrosis factor-*α*, interleukin-1*β*, and inducible NO synthase, when administered in pregnant female rats 2 h before the administration of endotoxin lipopolysaccharide [112]. Intraperitoneal treatment of pregnant rats with NAC (50 mg/kg) attenuated LPS-induced expression of inflammatory mediators in fetal rat brains and prevented postnatal hypomyelination [112]. NAC administered to pregnant rats in their drinking water (500 mg/kg/day), from E17 to birth, prevented LPS-induced oxidative stress in the hippocampus of male fetuses and restored long-term potentiation in the hippocampus and improved spatial recognition performance in male off-spring [113]. Posttreatment with NAC, 4 hours after the LPS challenge, prevented loss of glutathione in hippocampus and improved spatial learning deficits [114]. Furthermore, NAC induced marked neuroprotection, associated with improvement of the redox state and inhibition of apoptosis after LPS-sensitized hypoxic-ischemic brain injury in neonatal rats [115]. After neonatal hypoxia-reoxygenation in piglets, NAC reduced cerebral oxidative stress with improved cerebral oxygen delivery and reduced caspase-3 and lipid hydroperoxide concentrations in cortex [116]. Similarly, hypoxia-reoxygenation-induced cortical hydrogen peroxidase was reduced with NAC therapy [117]. In fetal sheep, NAC exacerbated LPS-induced fetal hypoxemia and hypotension and induced polycythemia [118]. In a randomized clinical trial on preterm newborns, therapy with NAC by continuous infusion during the first 6 days after birth did not change the incidence of periventricular leukomalacia or intraventricular hemorrhage, in the treated babies [119]. In addition, NAC is also associated with adverse reactions that limit its use in humans, particularly anaphylactic reactions, including rash, pruritus, angioedema, bronchospasm, tachycardia, and hypotension, usually occurring within 2 hours of the initial infusion [120].

## 11. Melatonin

Melatonin is the main product of the pineal gland, with high antioxidant and anti-inflammatory properties [121, 122] and is synthesized starting from tryptophan [123]. When synthesized, it is quickly released in all biological fluids such as bile, cerebrospinal fluid [124], blood, saliva [125], semen [126], and amniotic fluid [127] and it functions as time-giver in the regulation of the circadian rhythm [128]. This rhythm in mammals is generated by an endogenous circadian master clock located in the suprachiasmatic nucleus of the hypothalamus. It participates in several neuroendocrine and physiological processes and is considered also a tissue factor, a paracoid, an autocoid, an antioxidant and sometimes a hormone depending on its physiological actions. Melatonin has a broad-antioxidant spectrum, a direct or indirect effect, and an anti-inflammatory property [129, 130]. It is particularly interesting for its ability to cross all physiological barriers [131] and to be widely distributed in tissues, cells, and subcellular compartments. Working as a direct antioxidant, it is able to scavenge dangerous FR, such as OH^*∙*^, O_2_^−^, H_2_O_2_, and ONOO−, and as an indirect one it induces the production of antioxidant enzymes, including glutathione peroxidase, glutathione reductase, glucose-6-phosphate dehydrogenase, and superoxide dismutase and increases the efficiency of mitochondrial electron transport [132–134].

Particularly its ability to scavenge the dangerous “^*∙*^OH” is much higher than other antioxidants including mannitol, glutathione, and vitamin E [135, 136]. Moreover, melatonin has no prooxidant effects unlike many other antioxidants and does not interfere with the thrombolytic and neuroprotective actions of other drugs [137, 138]. In addition melatonin can inhibit the expression of adhesion molecules therefore curbing PMN infiltration and tricking the cascade of inflammation [121]. Moreover melatonin may inhibit the NF-*κ*B expression, which is a nuclear transcription factor involved in inflammation development by interacting with its receptor MT1. Blocking NF-*κ*B melatonin acts as an anti-inflammatory molecule through the inhibition of the biochemical events following NF-*κ*B activation, such as the NO and prostaglandins production by iNOS and COX-2 [139]. Melatonin could be a useful drug in preterm delivery, a condition highly susceptible to OS injury due to the need of oxygen use for neonatal resuscitation and to the immaturity of the antioxidant systems. Because of its lipophilic properties, melatonin easily crosses most biological cell membranes, including the placenta and the blood-brain barrier. Unfortunately, despite its anti-inflammatory and antioxidant functions, melatonin is not currently available for neonatal therapeutic trials and a neonatal intravenous formulation needs to be developed. It is still unclear how much melatonin is absorbed after oral or rectal doses in infants who have been cooled and asphyxiated, but when it was used as a compassionate drug in neonatal asphyxia, preliminary findings supported the possibility for its wider evaluation in perinatal medicine [140]. Other studies found also that melatonin administration after HI in immature rats has an excellent and long-lasting benefit on ischemic outcomes and could represent a potentially safe approach to perinatal brain damage in humans [141]. Post ischemia intraperitoneal administration of melatonin significantly protected the brain from injury and reduced infarct volume, mainly in the hippocampus and cerebral cortex. Melatonin treatment also improved functional recovery into adulthood [141]. The optimal neuroprotective dose still needs to be determined, although a 5-mg/kg infusion for 6 hours started 10 minutes after resuscitation and repeated at 24 hours augmented hypothermic neuroprotection in the newborn piglet [121].

Even if there are no studies on antenatal administration of melatonin, animal studies indicate that even doses as high as 200 mg/kg for several days during pregnancy in rats do not have toxic effects on either mother or fetus [142]. When administered directly to the sheep fetus after umbilical cord occlusion, melatonin attenuated the production of 8-isoprostanes and reduced the number of activated microglia cells and terminal deoxynucleotidyl transferase dUTP nick end labeling- (TUNEL-) positive cells in the brain [121]. In addition maternal administration prevented subsequent increase in free radical production in fetal sheep exposed to intrauterine asphyxia [143]. Maternal melatonin administration at the end of pregnancy reduced signs of cerebral inflammation and apoptosis after birth asphyxia in the spiny mouse [144]. When given in low doses (0.005–5 mg/kg i.p.), melatonin reduced brain lesions in the white matter >80%, and melatonin was still (but less) effective when given 4 hours after the insult and reduced learning deficits [145]. It was also shown how melatonin protected the cerebral white matter after 2 hours of hypoxic insults in newborn rats [146], decreased microglial activation and astroglial reaction, and promoted oligodendrocyte maturation in growth restricted rat pups [147]. Finally a recent observational study showed that preterm and term newborn infants are deficient in melatonin levels, and it is now being trialed daily for 7 days after premature birth to identify whether it will reduce the risk of prematurity-associated brain injury (MINT; ISRCTN15119574, 2014). Another investigation is underway in premature and full-term babies to identify optimal treatment doses. An Australian study evaluating melatonin to prevent brain injury in unborn growth-restricted babies is ongoing in which mothers receive melatonin during pregnancy and OS is monitored in maternal serum, placenta, and umbilical cord blood (NCT01695070, 2014). Based on all these data, melatonin is a well-documented multifunctional molecule that may be a useful therapeutic agent for the treatment of neonatal hypoxic-ischemic encephalopathy. Melatonin is safe, nontoxic, and available in pure form for human use. These results of animal experimental models and human case reports provide fundamental information on the need of and the potential usefulness of clinical trials to evaluate melatonin as a neuroprotective drug.

## 12. Lutein

Lutein belongs to the family of carotenoids. Studies conducted both in vitro and in vivo have identified several properties of lutein, showing a defensive action that occurs through the neutralization of FR and singlet oxygen. Studies report that lutein is able to reduce the risk of developing some ocular diseases or slow down their progression [148]. Lutein may play a role in tissues defense through a functional mechanisms using the phenomenon of deactivation (quenching) of singlet oxygen and of reactive oxygen species [149], protecting the retina from ischemic/hypoxic damage by its antioxidative, antiapoptotic, and anti-inflammatory properties [150]. Lutein and its isomer zeaxanthin in the macular pigment may play an important role in protecting the eyes of the newborn from the damage of light thanks to their ability to absorb blue light and their antioxidant action [151]. Lutein administration in newborns increases the levels of biological antioxidant potential (BAP), decreasing OS markers levels in healthy term newborns, suggesting potential for its testing in clinical trials to protect newborns from perinatal OS [152, 153]. There was also a difference between breastfed and formula fed infants. Breastfed infants had higher mean serum lutein concentrations than infants who consumed formula unfortified with lutein. These data suggested that approximately four times more lutein is needed in infant formula than in human milk to achieve similar serum lutein concentrations among breastfed and formula fed infants [154]. Manzoni et al. found no treatment-related adverse effect in 229 preterm infants supplemented with lutein. They found no significant differences in the threshold of ROP between treated versus not treated infants [155]. The same occurred for NEC and BPD. Interestingly they found that the progression rate from early ROP stages to threshold ROP was decreased by 50% [155] showing how lutein/zeaxanthin supplementation in preterm infants is well tolerated and can interfere with ROP progression. Rubin et al. assessed lutein safety and they demonstrated that supplemented infants had lower plasma C-reactive protein and that plasma lutein levels correlated with the full field electroretinogram-saturated response amplitude in rod photoreceptors in a cohort of 203 preterm newborns. Finally the supplemented group also showed greater rod photoreceptor sensitivity [156]. All these data suggest that lutein may be a promising drug against oxidative injury.

## 13. Dietary Supplements

Dietary supplements may also be promising in neuroprotection. Pomegranate juice is rich in polyphenols that was proven to protect the neonatal mouse brain against an HI insult when given to mothers in their drinking water [157]. A substantial protection in hippocampus, cortex, and striatum was observed even when given after the insult to neonatal animals [158]. Omega-3 polyunsaturated fatty acid supplementation can reduce brain damage and improve long-159 neurologic outcomes even 5 weeks after an HI insult to rodents [159].

## 14. Conclusions

Even if there are still gaps in knowledge about antioxidants and their role in neuroprotection, progress has been made in this field. Probably a combination of these drugs and supplements should be more extensively investigated in order to reduce brain injury to the developing neonate. The relationship between FR production and brain injury is complex. Clearly FR damage results from hypoxia, ischemia-reperfusion, neutrophil and macrophage activation, Fenton reaction, endothelial cell xanthine oxidase, phospholipid metabolism, nitric oxide, mitochondrial oxidative metabolism impairment, and proteolytic pathways. Each step in the oxidative injury cascade has become a potential target for neuroprotective intervention. The administration of antioxidants for suspected or proven brain injury is still not accepted for clinical use due to uncertain beneficial effects when treatments are started after resuscitation of an asphyxiated newborn. The challenge for the future is the early identification of high-risk babies to target a safe and not toxic antioxidant therapy in combination with standard therapies to prevent brain injury and long-term neurodevelopmental impairment.
